# Combined Bezafibrate and Medroxyprogesterone Acetate: Potential Novel Therapy for Acute Myeloid Leukaemia

**DOI:** 10.1371/journal.pone.0008147

**Published:** 2009-12-07

**Authors:** Farhat L. Khanim, Rachel E. Hayden, Jane Birtwistle, Alessia Lodi, Stefano Tiziani, Nicholas J. Davies, Jon P. Ride, Mark R. Viant, Ulrich L. Gunther, Joanne C. Mountford, Heinrich Schrewe, Richard M. Green, Jim A. Murray, Mark T. Drayson, Chris M. Bunce

**Affiliations:** 1 School of Biosciences, University of Birmingham, Birmingham, United Kingdom; 2 Henry Wellcome Building for Biomolecular NMR Spectroscopy, CRUK Institute for Cancer Studies, University of Birmingham, Birmingham, United Kingdom; 3 Division of Cancer Sciences and Molecular Pathology, University of Glasgow, Glasgow, United Kingdom; 4 Department of Developmental Genetics, Max-Planck Institute for Molecular Genetics, Berlin, Germany; 5 Institute of Medical Genetics, Charité-University Medicine Berlin, Berlin, Germany; 6 Centre for Clinical Haematology, Queen Elizabeth Hospital, Birmingham, United Kingdom; 7 Division of Immunity and Infection, University of Birmingham, Birmingham, United Kingdom; Dalhousie University, Canada

## Abstract

**Background:**

The majority of acute myeloid leukaemia (AML) patients are over sixty years of age. With current treatment regimens, survival rates amongst these, and also those younger patients who relapse, remain dismal and novel therapies are urgently required. In particular, therapies that have anti-leukaemic activity but that, unlike conventional chemotherapy, do not impair normal haemopoiesis.

**Principal Findings:**

Here we demonstrate the potent anti-leukaemic activity of the combination of the lipid-regulating drug bezafibrate (BEZ) and the sex hormone medroxyprogesterone acetate (MPA) against AML cell lines and primary AML cells. The combined activity of BEZ and MPA (B/M) converged upon the increased synthesis and reduced metabolism of prostaglandin D_2_ (PGD_2_) resulting in elevated levels of the downstream highly bioactive, anti-neoplastic prostaglandin 15-deoxy Δ^12,14^ PGJ_2_ (15d-PGJ_2_). BEZ increased PGD_2_ synthesis via the generation of reactive oxygen species (ROS) and activation of the lipid peroxidation pathway. MPA directed prostaglandin synthesis towards 15d-PGJ_2_ by inhibiting the PGD_2_ 11β -ketoreductase activity of the aldo-keto reductase AKR1C3, which metabolises PGD_2_ to 9α11β-PGF_2α_. B/M treatment resulted in growth arrest, apoptosis and cell differentiation in both AML cell lines and primary AML cells and these actions were recapitulated by treatment with 15d-PGJ_2_. Importantly, the actions of B/M had little effect on the survival of normal adult myeloid progenitors.

**Significance:**

Collectively our data demonstrate that B/M treatment of AML cells elevated ROS and delivered the anti-neoplastic actions of 15d-PGJ_2_. These observations provide the mechanistic rationale for the redeployment of B/M in elderly and relapsed AML.

## Introduction

Acute myeloid leukaemia (AML) is a devastating cancer characterised by the uncontrolled proliferation, abnormal survival and arrested maturation of leukaemic cells within the bone marrow. The rapid expansion of the leukaemic clone reduces haemopoiesis with loss of normal functioning neutrophils, platelets, and erythrocytes. If untreated, most patients die from infection, bleeding and/or anaemia within weeks of diagnosis.

Current best treatments utilise anthracyclines e.g. daunorubicin or idarubicin, alongside the pyrimidine and purine analogue cytarabine with or without 6-thioguanine [1], [2]. These drugs non-selectively inhibit DNA and RNA synthesis and consequently their anti-leukaemic activity is associated with high levels of systemic toxicity, including further reduction of haemopoiesis. Although the current therapies of choice, these agents fail to cure more than two thirds of those patients deemed able to tolerate the therapy [2], [3], [4]. The problem is further exacerbated by the molecular heterogeneity underlying the disease as well as its distribution within the population. AML incidence increases with age and >75% of patients are older than 60 years of age at diagnosis. These older patients have a much reduced capacity to tolerate high dose chemotherapy and their leukaemia's are associated with higher frequencies of unfavourable prognostic factors [5]. As a result, overall survival rates amongst this cohort are dismal with little improvement having been made over the last 20 years [2], [3], [4], [6], [7]. This lack of progress coupled with the frail nature of these patients presents them and their clinicians with limited therapeutic options. The majority of elderly patients receive supportive care alone or with non-intensive therapy [4], [8]. A review of 36 AML studies involving a total of 12,370 patients (median age 70 yrs) found that the median overall survival for patients receiving supportive care alone was only 7.5 weeks and for those receiving supportive care with non-intensive therapy only 12 weeks [5].

Knowledge of the molecular pathogenesis of AML and other leukaemia's has led to attempts to develop specific targeted agents and a number of these are now in clinical trial [2], [4], [9]. However, with the notable exception of all-trans retinoic acid (ATRA) in a subset of AML known as acute promyelocytic leukaemia (APL)[10], and imatinib mesylate (Glivec) and its derivatives in chronic myeloid leukaemia (CML)[11], few of these therapies have as yet had a large scale impact. This has lead to trials using combinations of targeted therapies [12]. However, the problem of developing targeted therapies for AML is complicated by the inherent heterogeneity and genetic complexity of the disease. Thus whilst attempts at new drug discovery remain important, their low success rates, long time scales and high costs impose serious limitations on progress for improving the outlook in this disease.

‘Drug redeployment’ provides an alternative treatment strategy that is gaining momentum across a broad spectrum of diseases [13], [14]. This approach tests the potential of established drugs in new disease settings. We and others have previously demonstrated the individual *in vitro* anti-proliferative and pro-differentiative actions of the sex steroid medroxyprogesterone acetate (MPA) and lipid regulating fibrate drugs against AML cell lines [15], [16], [17], [18], Burkitts lymphoma (BL) cells [19] and chronic lymphocytic leukaemia (CLL) cells [20]. Here we demonstrate improved combinatorial activity of bezafibrate (BEZ) and MPA (B/M) against AML cell lines and primary AML cells. We demonstrate that the activity of the drugs when combined remains selective against AML cells over normal myeloid blasts. Furthermore we demonstrate that the antitumor activity of B/M against AML cell differs from the activity of the same drugs against CLL cells in that it converges on the increased synthesis and decreased metabolism of prostaglandin D_2_ (PGD_2_) and its potently anti neoplastic derivative 15deoxyΔ^12, 14^ prostaglandin J_2_ (15d-PGJ_2_).Several studies have identified potent activities of 15d-PGJ_2_ against diverse cancers [21], [22], [23], [24], [25], [26], [27], [28], [29]. The cyclopentenone configuration of this prostaglandin renders it highly reactive facilitating non-enzymatic covalent bonding to thiol residues in multiple biological substrates [30], [31], [32], [33], [34], [35], [36], [37], [38]. The result is modulation of the activity and/or levels of multiple cellular targets, the spectrum of which will be cell context specific but which most likely explain the broad anti tumour activity of 15d-PGJ_2_. Given its bioactive nature, administration of 15d-PGJ_2_ is likely to be associated with high toxicity and low bioavailability due to conjugation of 15d-PGJ_2_ to extracellular targets, including serum proteins [39]. An improved strategy is therefore to promote the accumulation of endogenously formed 15d-PGJ_2_ within target cells. Since 15d-PGJ_2_ arises non-enzymatically from PGD_2_ [40], [41] the elevated synthesis of this prostanoid is in turn dependent on the elevation of PGD_2_. We demonstrate here that in the case of AML, elevation of PGD_2_ and 15d-PGJ_2_ with associated anti-leukaemic activity can be achieved with drugs that are available, relatively cheap and familiar in the clinical setting.

## Results

### The Anti-Leukaemic Actions of BEZ and MPA against AML Cell Lines Are Most Potent When Combined

**Figure 1:**
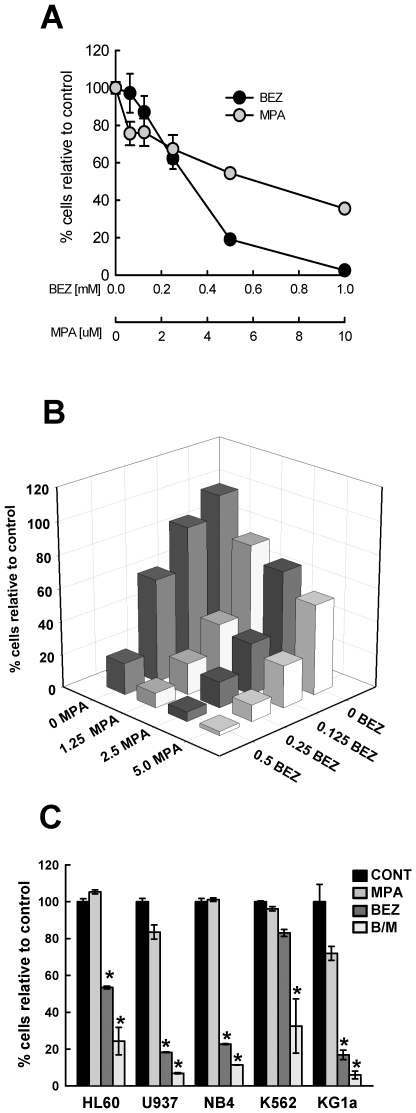
Dose dependent killing by BEZ, MPA and B/M of myeloid cell lines. Cell viability as % of solvent treated controls was determined in KG1a cells by Cell-Titre Blue assay following 10 days treatment with (**A**) increasing doses of MPA or BEZ alone, or (**B**) cross-titrations of BEZ (mM) and MPA (µM). Data shown is mean of N = 3 experiments (**C**) 5 different myeloid cell lines were treated with either solvent control, 0.5 mM BEZ, 5 µM MPA or the combination (B/M) for 7 days. Cell viability was calculated for treatments relative to solvent treated controls after readings had been adjusted for feeding regimens over the 7 days of treatment. Mean±sem is shown for 5 cell lines from a minimum of N = 3 experiments. Individual datapoints are shown in Figure S1. Statistics * p<0.01.

Figure 1A shows the dose dependent killing of KG1a cells by MPA and BEZ and illustrates the greater individual potency of BEZ. After 10 days treatment with 0.5 mM BEZ cell viability had been reduced to 20% of controls whereas 5 µM MPA had reduced cell viability to just 60% of controls. In BEZ and MPA cross titration experiments, potentiation of killing of KG1a cells was clearly evident with near total loss of viability with 0.5 mM BEZ and 5 µM MPA after 10 days (Figure 1B). Similar results were also observed in HL60 and K562 cells (data not shown) and are consistent with our previous studies in Burkitt's lymphoma cells [19]. Consequently, all subsequent experiments were performed with 0.5 mM BEZ and 5 µM MPA both when alone and in combination (B/M). The anti-proliferative effects of BEZ and MPA on a panel of cell lines representing diverse forms of AML including U937, NB4 (PML:RARα^+ve^ APL), HL-60, K562 (BCR:ABL^+ve^ AML blast crisis of chronic myeloid leukaemia) and KG1a cells are shown in Figure 1C and Figure S1. As in KG1a cells, BEZ was the more potent antiproliferative individual agent in U937, NB4 and HL60 cells although neither agent alone was particularly effective against K562 cells. However, B/M treatment was the most potent (p<0.01) at reducing cell number across all the cell lines examined.

### BEZ and MPA Variably Induces Differentiation and Apoptosis in Myeloid Cell Lines

**Figure 2:**
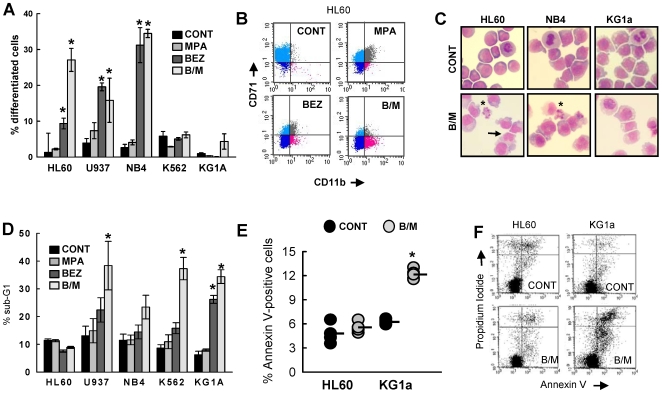
Cellular consequences of MPA and BEZ treatment. (**A**) Differentiation was measured by flow-cytometry using the myeloid differentiation antigen CD11b for HL60, NB4, U937, KG1a cells and the erythroid antigen Glycophorin-A for differentiation of K562. Mean data±sem from a minimum of N = 3 experiments are shown. All datapoints are shown in Figure S2A. (**B**) Representative dotplots for HL60 cells following 7 days treatment stained both with CD11b and the proliferation marker CD71. Pale blue shows non-differentiated cells (CD71+, CD11b−), dark blue cells that have lost CD71 (CD71−, CD11b−), and pink identifies differentiated neutrophils (CD71−, CD11b+). (**C**) Cell morphology was analysed by Jenner-Giemsa staining of cytospin preparations of cells treated for 7 days. Apoptotic cells are highlighted by a star and differentiating neutrophils by an arrow. (**D**) % Sub-G1 events were measured by cell cycle analysis of propidium iodide stained cells following 7 days treatment. Mean data±sem for 5 cell lines from a minimum of N = 3 experiments are shown. All datapoints are shown in Figure S2B. (**E**) HL60 and KG1a cells were treated with either solvent CONT or B/M for 48 hours. Apoptosis was assessed by incubation with annexin-V and propidium iodide followed by flow cytometry. Individual datapoints from a minimum of N = 4 are shown with a black bar indicating the mean. (**F**) Representative dotplots of annexin V and propidium iodide stained HL60 and KG1a cells following treatment with either solvent CONT or B/M for 48 hours. Statistics *p<0.01.

The myeloid cell surface antigen CD11b was used in flow cytometry to assess differentiation of U937, NB4, HL60 and KG1a cells. Erythroid differentiation of K562 cells was measured by glycophorin A (Gly A) expression. Consistent with our earlier findings in HL60 cells, MPA alone caused little or no differentiation in any of the cell lines (Figure 2A & Figure S2A) whereas BEZ induced differentiation of U937, NB4 and HL60 cells to varying degrees. Combined B/M treatment induced markedly increased CD11b expression in HL60 cells compared to the individual agents. The combinatorial action of B/M upon HL60 differentiation was confirmed by the reciprocal loss of the more primitive cell marker CD71 (Figure 2B). B/M combined treatment also induced differentiation of U937 and NB4 cells but in these cells there was no increase in B/M over BEZ alone (Figure 2A & Figure S2A). In marked contrast MPA, BEZ and B/M failed to induce either the erythroid or myeloid differentiation of K562 or KG1a cells respectively. This was confirmed by analyses of cell morphology. As shown in Figure 2C, B/M treated NB4 and HL60 cultures clearly contained maturing neutrophils whereas maturing cells were not evident in B/M treated KG1a cultures.

Cell cycle analyses at day 7 identified a marked G1 cell cycle arrest in those cell lines that displayed differentiation in response to B/M (data not shown). In K562, KG1a and U937 but not HL-60 and NB4 cells, cell cycle analyses further identified the accumulation of a sub-G1 fraction, indicative of post apoptotic cells (Figure 2D & Figure S2B). These data indicated that the cell responses to B/M treatment were varied, ranging from strong cell cycle arrest and differentiation to apoptosis. A more detailed comparison of KG1a and HL60 cells confirmed induction of annexin-V labelling (Figure 2E) together with uptake of propidium iodide (PI), consistent with apoptosis in KG1a cells but not in HL60 cells (Figure 2E and 2F).

### In Vitro Anti-Leukaemic Activities of BEZ and MPA Are Recapitulated in Primary AML Cells but Not Normal CD34^+ve^ Cells

**Figure 3:**
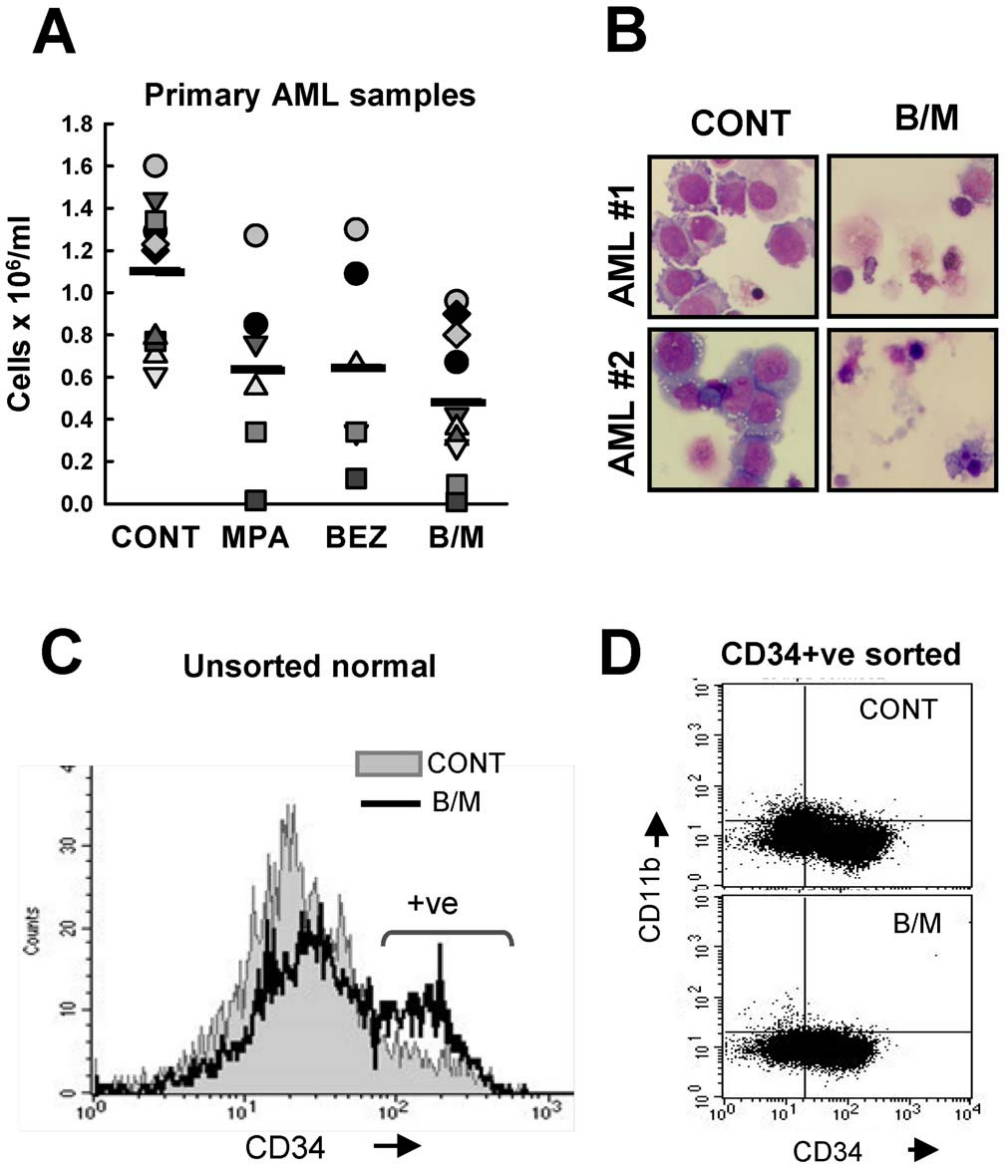
BEZ and MPA also kill primary AML cells but not normal myeloid progenitors. (**A**) Primary AML cells were treated for 5 days. Cell numbers were determined by manual counts and are presented as number of cells×10^6^/ml at day 5. The plot shows summary data for 9 individual AMLs and the mean identified by bars. (**B**) Cell morphology for 2 representative AML samples are shown as determined by Jenner-Giemsa staining of cytospin slides. (**C**) Non-sorted mobilised peripheral blood cells were treated with solvent CONT or B/M. Histogram shows representative CD34 staining after 8 days treatment. (**D**) CD34+ve purified normal mobilised cells were treated with either solvent CONT or B/M for 8 days. The dotplots shown are CD34 and CD11b expression following treatment.

Primary AML cells also demonstrated *in vitro* sensitivity to BEZ, MPA and B/M (Figure 3A). At day 8, control cultures originally plated at 1×10^6^ cells/ml contained 1.1±0.13×10^6^ cells/ml. In contrast B/M treated cultures contained 0.52±0.11×10^6^ cells/ml (p<0001). Morphological examination of primary AML cultures (not shown) and cytospins (Figure 3B) prepared from B/M treated AML cells indicated that B/M induced loss of cells was most frequently mediated by cell killing rather than overt differentiation.

In marked contrast, treatment with BEZ, MPA or B/M had little effect on normal myeloid progenitors (CD34+ve cells). Unsorted mobilised mononuclear cells which are enriched with CD34+ve myeloid progenitors were treated for 8 days. No changes in cell number were recorded in response to either drug alone and only a small decrease was observed following B/M treatment. Despite this decrease, the proportion of viable CD34+ve cells increased, indicating no significant loss of these cells (Figure 3C). Similarly, treatment of purified mobilised normal donor CD34+ve cells with BEZ, MPA and B/M did not result in apoptosis or frank differentiation. After 8 days, B/M treated cultures contained 89% of the number of viable cells in untreated cultures. Furthermore, although a slight diminution of CD34 fluorescence intensity was observed, overt differentiation as measured by the maturation marker CD11b was not induced (Figure 3D).

### MPA Targets AKR1C3 in AML Cells

**Figure 4:**
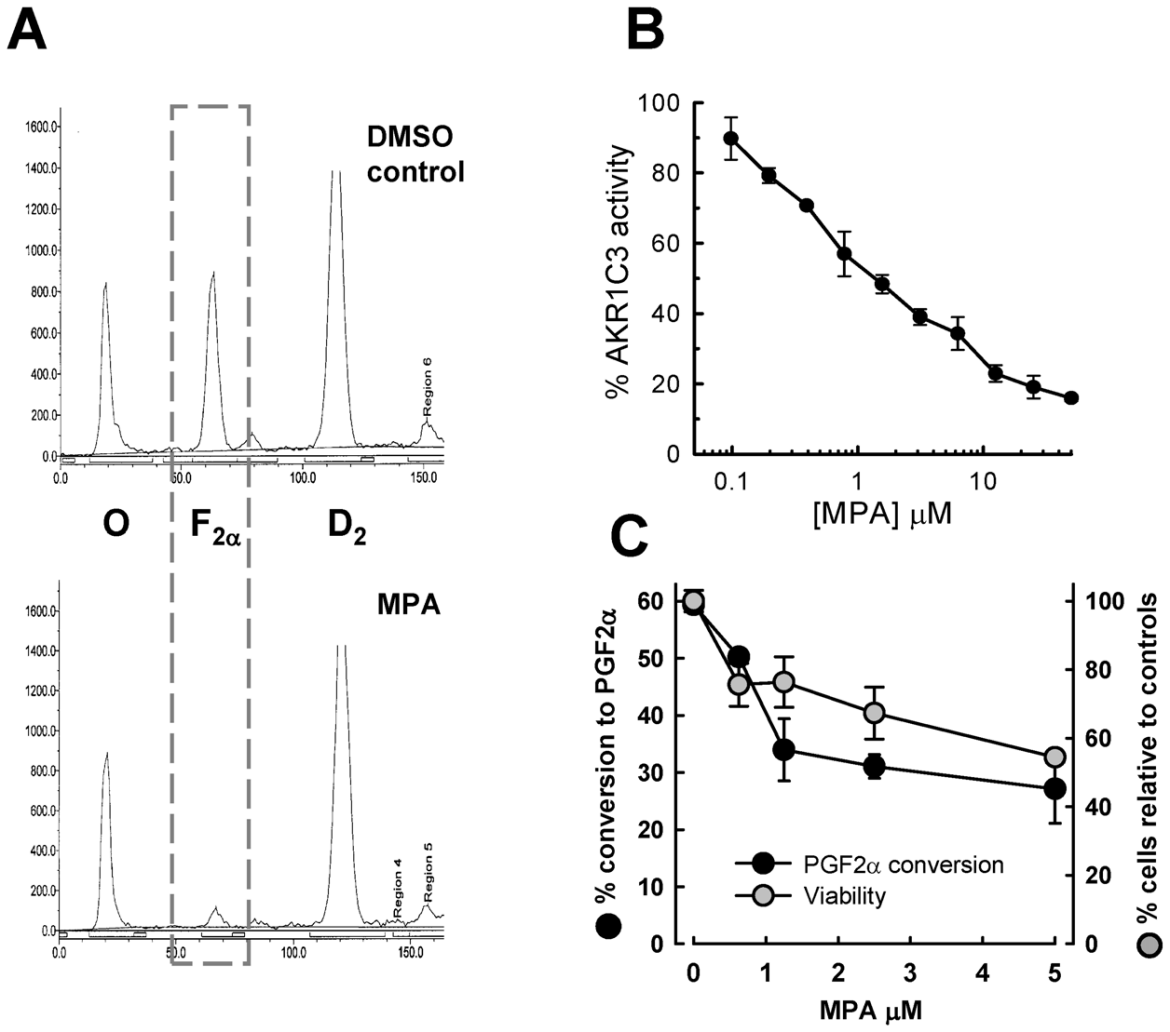
MPA inhibits AKR1C3 PGD_2_ 11β keto-reductase activity. (**A**) ^3^H-PGD_2_ turnover was determined by thin layer chromatography (TLC) on KG1a cells treated with either solvent control or 5 µM MPA. Representative TLC traces are shown from a minimum of N = 4 experiments. Abbreviations; O = origin, F_2α_  = 11β-PGF_2α_, D_2_ = PGD_2_. (**B**) Inhibition of recombinant AKR1C3 protein activity by MPA. The plot shows percentage of AKR1C3 activity in the presence of increasing concentrations of MPA relative to AKR1C3 in the absence of MPA. The data are means±s.d. from a single experiment performed in triplicates. (**C**) Conversion of ^3^H-PGD_2_ to 9α-11β-PGF2α was determined by TLC in KG1a cells treated with MPA titrations and compared to cell viability at day 10. Mean data±sem from a minimum of N = 3 are shown.

We and others have shown AML cells to express the aldo-keto reductase AKR1C3 [42], [43], [44] and our rationale for selecting MPA for redeployment as a potential antileukaemic drug is as an inhibitor of this enzyme [28]. However, although MPA had been shown to inhibit other aldo-keto reductases [45], it had not been directly demonstrated as an inhibitor of AKR1C3. Like other members of the AKR1C subfamily of aldo-keto reductases, AKR1C3 displays substrate promiscuity [46] but amongst the human sub-family possesses unique PGD_2_-11-ketoreductase activity generating 11β-PGF_2α_ from PGD_2_. Our previous studies have shown that this activity is prominent in AML cells but not detectable in CLL cells [44], [20]. MPA inhibited both the cellular AKR1C3 activity in KG1a myeloid cells, as measured by the decreased conversion of ^3^H-PGD_2_ to ^3^H-11β-PGF_2α_ (Figure 4A), and the *in vitro* activity of recombinant-AKR1C3 protein (IC_50_ = 1.1 µM; Figure 4B). In dose response experiments performed in KG1a cells, the inhibition of AKR1C3 mediated PGD_2_ conversion to 11β-PGF_2α_ mirrored the dose response of reduced cell viability (Figure 4C). Together these data and those from our previous study that identify AKR1C3 as having PGD_2_- 11-keto reductase activity in AML cell lines [44] support our model of the action of MPA against AML cells.

### The Anti-Leukaemic Activities of BEZ Are Associated with ROS Generation

**Figure 5:**
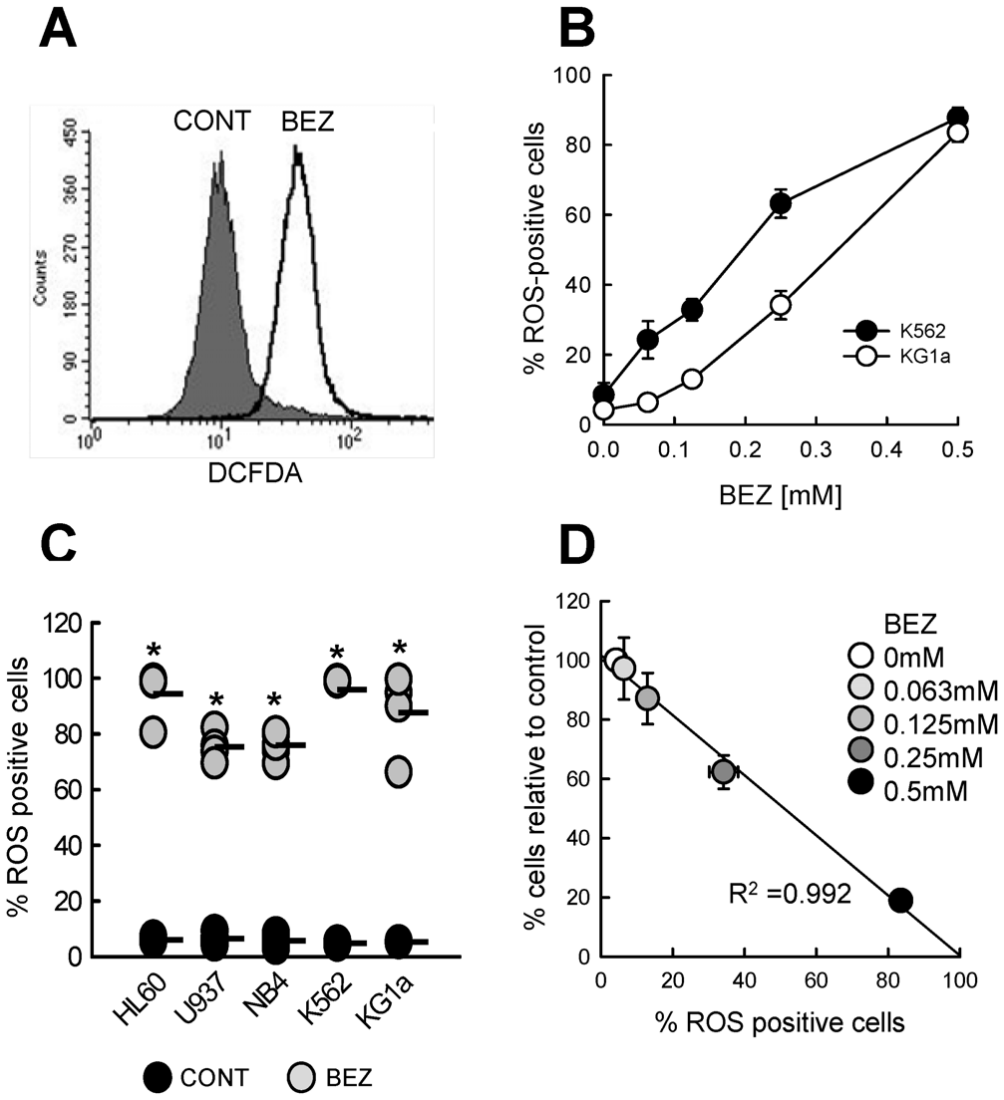
BEZ induction of ROS is concentration dependent and correlates with cell killing. BEZ induced reactive oxygen species (ROS) were measured at 14 hours by staining with carboxy-H_2_DCFDA and analysis by flow cytometry. (**A**) Histogram shows representative results from HL60 cells. (**B**) ROS induction was measured in K562 and KG1a cells after 14 hrs treatment with different BEZ concentrations (mean±sem from N = 4 experiments). (**C**) ROS induction with 0.5 mM BEZ for 5 myeloid cell lines is shown from a minimum of N = 3 experiments for each cell line. Mean is indicated by black bar. (**D**) % viable KG1a cells at day 10 compared to controls plotted against the % cells positive for ROS at increasing BEZ concentrations. Statistics *p<0.01.

The original rationale made by ourselves and others for the redeployment of fibrates as anti-AML agents, was based on their activity as ligands for the nuclear receptor PPARα. However, the required BEZ concentrations observed here suggest an alternative mechanism. BEZ has previously been shown to induce oxidative stress in cells [47], [48]. In keeping with this, BEZ treatment of K562 and KG1a cells induced a rapid (within 2 hours, data not shown) and sustained generation of reactive oxygen species (ROS) that was BEZ concentration dependent (Figure 5A and 5B). Similar induction of ROS was observed in all AML cell lines when treated with 0.5 mM BEZ (Figure 5C). Finally, using the example of KG1a cells, we observed that cell killing by BEZ tightly correlated with the percentage of ROS positive cells (Figure 5D).

### The Actions of BEZ and MPA on AML Cells Converge on the Accumulation of PGD_2_ and Its Downstream Reactive Product 15d-PGJ_2_

**Figure 6:**
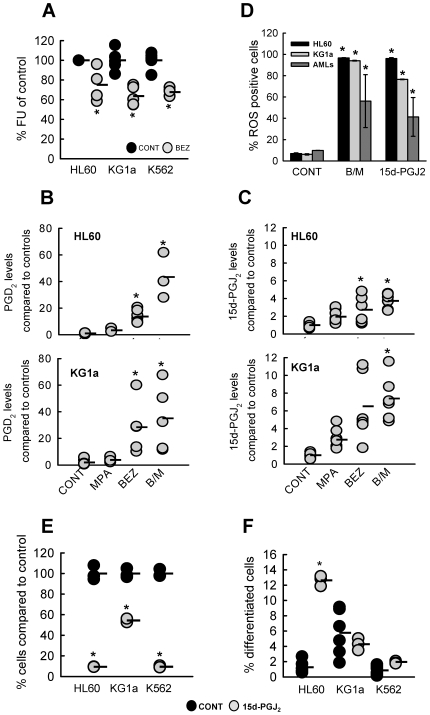
BEZ and MPA modulate PGD_2_ and 15d-PGJ_2_ levels. (**A**) 0.5 mM BEZ induced lipid peroxidation was measured by treating cells labelled with the naturally fluorescent lipid *cis*-parinaric acid. Data is presented as % fluorescence units (FU) compared to solvent control cells for N = 5 experiments for each cell line. Mean is indicated by black bar. (**B & C**) Endogenous levels of (**B**) PGD_2_ and (**C**) 15d-PGJ_2_ were determined in HL60 and KG1a cells by ELISA following 2 hours treatment. Prostaglandin levels are shown relative to untreated controls for a minimum of 4 experiments. Mean is indicated by black bars. Statistics: * p<0.05. (**D**) ROS induction was determined by staining with DCFDA and flow cytometry in HL60, KG1a and primary AML samples (n = 3) after 48 hours treatment with CONT, B/M or 10 µM 15d-PGJ_2_. Data shown are mean±sem. Individual datapoints are shown in Figure S4. (**E & F**) HL60, K562 and KG1a cells were treated for 7 days with either solvent control or 5 µM 15d-PGJ_2_. Data is shown from a minimum of N = 3 experiments. Mean is indicated by black bar. (**E**) Cell viability was assessed by Alamar blue and calculated as % of control cells. (**F**) Differentiation was assessed by CD11b staining of HL60 and KG1a and GlyA staining of K562 cells followed by flow cytometry. Statistics: * p<0.01.

PGD2 is synthesised from arachidonic acid by both a cyclooxygenase (COX)-dependent pathway [49] and COX-independent, an oxidative stress-dependent, lipid peroxidation-isoprostane pathway [50] (Figure S3). Given that the lipid peroxidation-dependent isoprostane pathway has been shown to be elevated in periods of oxidative stress [51], we postulated that BEZ-induced oxidative stress would result in lipid peroxidation. Consistent with this, 0.5 mM BEZ treatment of AML cell lines labelled with the naturally fluorescent lipid cis-parinaric acid was associated with a significant decrease in fluorescence, indicative of increased lipid peroxidation (Figure 6A). We therefore reasoned that the effects of BEZ and MPA may converge on the elevation of cellular PGD_2_ via increased lipid peroxidation-mediated synthesis and decreased metabolism by AKR1C3. We also reasoned that as a consequence of increased PGD_2_ levels, the levels of its potently anti-neoplastic dehydration product 15d-PGJ_2_ would also be elevated_._ Indeed, treatment with MPA or BEZ alone and B/M resulted in increased levels of both PGD_2_ and 15d-PGJ_2_ in HL60 and KG1a cells (Figure 6B & C). MPA induced 3.3±0.9 fold and 3.8±0.9 fold increases in PGD_2_ in HL60 and KG1a, respectively as compared to solvent treated control cells (Figure 6B). As may be expected of an inducer of lipid peroxidation and therefore increased isoprostane pathway activity, the effects of BEZ were greater than MPA with 13.7±1.6 and 28.4±9.9 fold increases in PGD_2_ in HL60 and KG1a, respectively (Figure 6B). B/M gave the most marked elevation especially in HL-60 cells with a 43.4±9.9 fold increase, whereas KG1a had a 35.1±12.2 fold increase. 15d-PGJ_2_ levels followed a similar trend in both cell lines (Figure 6C).

It has been demonstrated that 15d-PGJ_2_ itself can potently induce ROS [21]. Consistent with this, treatment of AML cell lines and primary AML cells with 15d^−^PGJ_2_ also resulted in the induction of ROS levels similar to those seen in response to B/M (Figure S4). Furthermore, treatment of HL60, K562 and KG1a cells with 15d-PGJ_2_ recapitulated the antiproliferative actions of B/M (Figure 6E) and the enhanced differentiation of HL-60 cells but not of K562 or KG1a cells (Figure 6F).

### Actions of BEZ and MPA Recapitulate Known Activities of 15d-PGJ_2_

The above observations suggested that the activities of B/M against AML cells may be largely mediated by the generation of 15d-PGJ_2._ Previous studies have identified multiple mechanisms by which 15d-PGJ_2_ exerts its anti-neoplastic activity. The nature and range of these actions suggests that the detailed effects of 15d-PGJ_2_ against individual tumours may show cell context specificity. We studied some of the previously reported activities of 15d-PGJ_2_ in AML cells following treatment with BEZ and MPA.

#### Glutathione depletion

**Figure 7:**
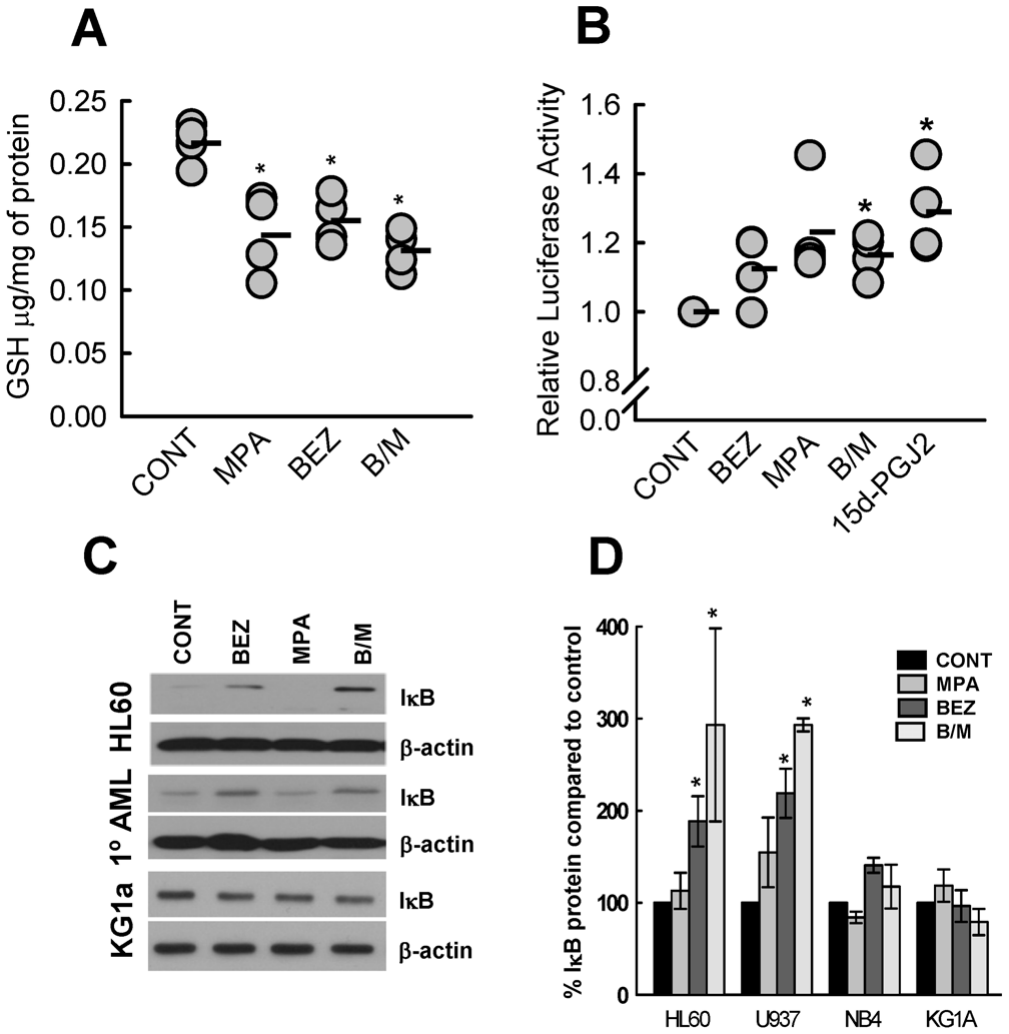
Downstream consequences of elevating 15d-PGJ_2_ levels. (**A**) Elevating 15d-PGJ_2_ levels results in lowering of reduced-glutathione (GSH). HL60 cells were treated for 48 hours and µg GSH per mg of protein measured. Data shown is from 4 experiments and the mean is indicated by a black bar. (**B**) PPARγ transcriptional activity was measured after 14 hours treatment of PPRE-luciferase reporter plasmid and pRenilla luciferase transfected HL60 cells with either MPA, BEZ, the combination (B/M) or 5 µM 15d-PGJ_2_ (PGJ_2_). Data shown is luciferase activity compared to control untreated cells for N = 5 experiments. Mean is indicated by black bar. (**C&D**) IκB levels were determined by western blotting of cells treated for 14 hrs. Levels were normalised for loading by β-actin westerns. (**C**) Representative western blot results for HL60, KG1a and a primary AML. (**D**) Graph represents mean±sem of densitometry performed on a minimum of N = 3 experiments for each cell line. All individual datapoints are shown in Figure S7. Statistics: * p<0.05.

The ratio and amounts of reduced glutathione (GSH) and oxidised glutathione (GSSG) are important in regulating the redox state of a cell. Under conditions of oxidative stress, GSH/GSSG levels become depleted and imbalanced, leading to cell differentiation or cell death. Importantly, 15d-PGJ_2_ concentrations that induce apoptosis and differentiation have been associated with depletion of cellular GSH [21], [52]. Furthermore, 15d-PGJ_2_ levels have been shown to be tightly regulated through GSH-conjugation and subsequent cellular export [53], [54], [55]. We therefore determined changes in GSH levels following treatment with BEZ, MPA and B/M. Significant decreases in both GSH (Figure 7A) and GSSG (Figures S5 & S6) levels were observed following drug treatment. In the case of GSH reduction the B/M effect was not greater than either drug alone whereas the greatest reduction in GSSG was observed in response to B/M (Figure S6).

#### PPARγ activation

Amongst the first biological activities ascribed to 15d-PGJ_2_ was as an activating ligand for the nuclear receptor PPARγ [56], [57]. Luciferase reporter assays in transiently transfected HL60 cells identified PPARγ-activation in response to MPA, BEZ and B/M (Figure 7B). Although small, the observed changes in PPARγ-activation mirrored those seen with 5 µM 15d-PGJ2 treatments.

#### IκB-accumulation

15d-PGJ_2_ has been shown to regulate NF-κB activity at multiple levels [30], [31]. This includes regulation of IκB (inhibitor of κB) protein levels by inhibiting the activity of the IKK (IκΒ-kinase) complex that targets IκB for ubiquitin-mediated protein degradation [30]. Inhibition of IκB-phosphorylation results in its accumulation and consequent inhibition of NF-κB transcriptional activity. Western blot analysis revealed IκB-accumulation in MPA, BEZ and B/M treated HL60 cells (Figure 7C & D, Figure S7). The pattern of IκB accumulation mirrored that of 15d-PGJ_2_ accumulation with MPA having the least or little effect, BEZ having a greater effect and the combination being most effective. Similar accumulations were observed in U937 cells and in a sample of primary AML cells, (Figure 7D) however KG1a and NB4 cells did not show an accumulation of IκB (Figure 7C&D, Figure S7). Since 15d-PGJ_2_ is known to regulate NF-κB transcriptional activity at multiple levels we cannot rule out the possibility that B/M may alter NF-κB in KG1a and NB4 cells by mechanisms other than accumulation of IκB.

### The Cellular Activities of BEZ and MPA Are Enhanced by Physiological Levels of Vitamin A and Vitamin D_3_

**Figure 8:**
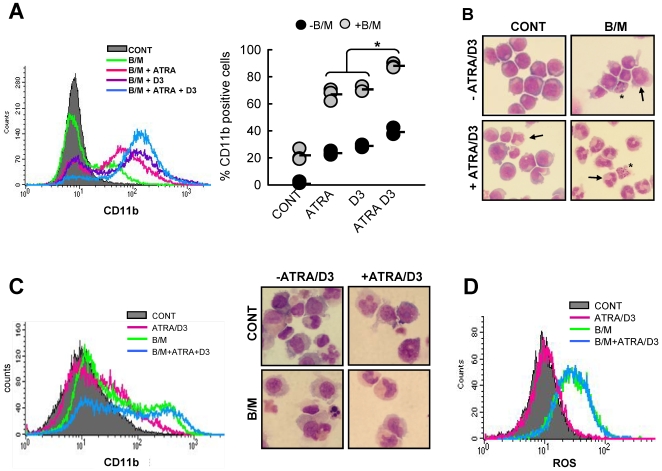
Physiological levels of ATRA and Vitamin D3 potentiate the actions of BEZ and MPA. (**A**) HL60 cells were treated for 7 days with solvent control, B/M alone and B/M combined with either 1 nM ATRA, 1 nM 1α,25(OH)_2_ vitamin D_3_ (D_3_) or both. A representative histogram of CD11b flow cytometry is shown in the *left panel* and results from N = 4 experiments in the *middle panel*. Mean is indicated by the black bar. (**B**) Cell morphology was analysed by Jenner-Giemsa staining of cytospins. Differentiated neutrophils are identified by classical poly-lobed nuclei (arrows) and apoptotic cells highlighted by asterix. Statistics: *p<0.001. (**C**) Primary AML cells taken from a karyotypically normal non-APL AML were treated with solvent CONT, B/M±1nmATRA/1nM D3. Differentiation was determined by CD11b staining and flow cytometry and by morphological analysis of Jenner-Giemsa stained cytospins after 18 days of treatment. (**D**) ROS generation was measured as described above in primary AML cells treated with cont, B/M±1nmATRA/1nM D3 for 48 hours.

When considering the translation of this study into a clinical trial we wished to better understand how to exploit these effects. Since we and others have previously shown that fibrates and MPA each separately enhance ATRA and 1α,25(OH)_2_ vitamin D_3_ (D3) induced HL-60 cell differentiation [15], [17], [58] we reasoned that where the combined action of B/M results in differentiation this may be reciprocally limited in the absence of ATRA and D3. In an attempt to mimic physiological levels of ATRA and D_3_ that might be expected in patients with adequate daily vitamin A and D intake, we treated HL60 cells with B/M together with 1 nM ATRA, 1 nM D_3_ and the combination (ATRA/D_3_). As in Figure 2A, B/M alone caused differentiation in ∼20% of HL60 cells, 1 nM ATRA+1 nM D_3_ (ATRA/D_3_) alone caused differentiation in ∼35% as measured by expression of the differentiation marker CD11b at day 7 (Figure 8A). However, morphologically relatively few cells had reached terminal neutrophil differentiation (Figure 8B). Addition of ATRA or D3 individually to B/M resulted in a significant increase in differentiated cells (Figure 8A). However, the addition of B/M together with ATRA/D_3_ resulted in ∼90% of cells expressing increased CD11b and morphologically all cells had differentiated into polymorphonuclear neutrophils and/or undergone apoptosis (Figure 8A & B) demonstrating strong complementary interactions between B/M and physiological levels of ATRA and D_3_. Prolonged survival and differentiation was rarely seen in primary AML cells however, when it did occur, we also observed complimentarity between B/M and ATRA/D3. This is illustrated in the primary AML (non-APL) sample shown in Figure 8C. CD11b expression was detected in 4.5% of control cells compared to 17.5% in ATRA/D3 treated cells. B/M alone induced CD11b expression in 32%, which was further increased to 46% when cells were treated with B/M & ATRA/D3 (Figure 8C). Interestingly, the addition of ATRA/D3 did not interfere with induction of ROS by B/M indicating that these agents were working by complementary actions (Figure 8D).

## Discussion

Drug redeployment has already demonstrated great promise in haemato-lymphoid malignancies including, thalidomide in myeloma [59] and valproic acid and arsenic trioxide in AML [60], [61]. However, few studies have sought to redeploy combinations of old drugs that deliver greater potency than either drug alone. We demonstrate here that exploiting some understanding of the mechanisms of drug actions against cancer cells allows the rational testing of such potentially beneficial combinations.

The anti-neoplastic activities of 15d-PGJ_2_ have been described in many reports [21]–[29], however few studies have sought strategies for utilising this potential. Although a novel cycloanthanilylproline derivative, Fuligocandin B, has been shown to induce 15d-PGJ_2_ production in treated cells the compound has yet to undergo toxicity testing in humans [62]. Here, we demonstrate that 15d-PGJ_2_ and its precursor, PGD_2_, can be elevated in AML cells using already available drugs with good safety profiles. BEZ treatment mediated sustained ROS generation with associated lipid peroxidation, downstream synthesis of PGD_2_ and consequently, generation of 15d-PGJ_2_. MPA, further elevated PGD_2_ and 15d-PGJ_2_ levels by inhibiting the metabolism of PGD_2_ by AKR1C3 (Figure S8).

15d-PGJ_2_ is a cyclopentenone prostaglandin. These prostaglandins have unsaturated α,β ketone moieties that allow non-enzymatic covalent modification of cellular targets [63]. It is this reactive nature that is thought to be responsible for their potent and wide-ranging properties [63], [64]. Proteomic approaches have identified the conjugation of 15d-PGJ_2_ with many protein targets including multiple components of mitochondria, the cytoskeleton and also transcriptional networks such as NF-κ
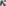
B [30]–[38]. Importantly, B/M treatment of AML cell lines and primary AMLs recapitulated some of the known anti-neoplastic activities of 15d-PGJ_2._ However these actions were not uniform across all the cell types tested. Similarly, we observed differential apoptotic and differentiation responses to B/M in both AML cell lines and primary AML cells. These variations may reflect the molecular heterogeneity of AML and the consequent contextual actions of 15d-PGJ_2_.

In contrast, 15d-PGJ_2_ also generated ROS in all AML cell lines and primary AML cells tested. It may therefore be that B/M treatment induces a cycle of direct and secondary 15d-PGJ_2_-mediated ROS generation thereby perpetuating a cycle of glutathione depletion and oxidative stress. The importance of ROS as well as 15d-PGJ_2_ in mediating the anti-leukaemic activity of B/M is highlighted by our recent NMR based metabolomics study of B/M treated AML cell lines [18]. It has been shown that ROS mediates the direct chemical conversion of α-ketoglutarate to succinate [65], [66], [67]. Notably, our metabolomics study identified drug mediated imbalances within the TCA cycle including the depletion of α-ketoglutarate and the accumulation of succinate [18]. This effect was recapitulated in cell extracts by the application of the ROS hydrogen peroxide. Therefore the antileukaemic actions of B/M against AML are complex and most likely mediated by separate and overlapping actions of both 15d-PGJ_2_ and ROS.

Although our studies have focused upon the accumulation of PGD_2_ and 15d-PGJ_2_ in B/M treated AML cells, it should not be ignored that part of the activity of MPA against AML cells may also be mediated by the diminution of 11β-PGF_2α_ (Figure S8) and future studies should address this possibility. Furthermore, since the anti-neoplastic activities of 15d-PGJ_2_ have been demonstrated in multiple tumour models [21]–[29], the potential of B/M based therapy may also extend beyond AML into other 15d-PGJ_2_ sensitive tumours. Our observations that B/M has greater efficacy against CLL, BL and now AML cells than either BEZ or MPA alone would appear to support this contention. However, it is important to note that although we have demonstrated that CLL cells express AKR1C3 we were unable to demonstrate AKR1C3 PGD_2_-11-ketoreductase activity in these cells [20]. These observations indicate that the substrate promiscuity of AKR1C3 is of real biological importance and has implications for its functions in different cell contexts. Hence, further studies are required to determine how B/M and particularly MPA or other AKR1C3 inhibitors exert anti tumour activities in different tumour settings.

In summary our findings support the investigation of B/M as novel therapy in elderly and relapsed AML. We have instigated such a trial, the results of which will be published in the near future. Given the low direct and supportive care costs of these agents, and their activity against multiple tumour types this study identifies an affordable potential anti-cancer therapy in developing countries where fiscal and other restraints limit the availability of conventional chemotherapy.

## Materials and Methods

### Reagents

Bezafibrate (0.5 M in DMSO), MPA (5 mM in ethanol), ATRA (1 mM in DMSO), and 1α, 25(OH)_2_ vitamin D_3_ (1 mM in ethanol) (Sigma Aldrich). PGD_2_ and 15dΔ-^12,14^PGJ_2_ (Affiniti, UK) were dissolved in DMSO to yield 20 µM stocks and stored at −20°C.

### In Vitro Recombinant AKR1C3 Activity Assay

Recombinant N-terminal His-tagged recombinant AKR1C3 was produced as previously described [68]. Enzyme reactions (1 ml-volumes) contained 15 µg recombinant AKR1C3, 4 µM phenanthrenequinone (Sigma, UK), 150 µM NADPH (Sigma) and 0–100 µM MPA in 50 mM potassium phosphate buffer (pH 6.5) at 35°C and measured as the rate of change of pyridine nucleotide absorbance at 340 nm.

### Primary Cells, Cell Lines and Treatments

HL-60, NB4, U937, K562 and KG1a myeloid cell lines were maintained in RPMI 1640 medium supplemented with 10% (v/v) heat-inactivated fetal bovine serum (FBS) and penicillin/streptomycin (Gibco, Invitrogen Ltd, UK). Unless specified otherwise, 1×10^6^ cells in 4 mls were treated with solvent control, 5 µM MPA, 0.5 mM BEZ or the combination B/M. 1 nM ATRA+1 nM Vitamin 1,25(OH)_2_ vitamin D_3_ (D3) (Sigma, UK), 10 µM PGD_2_ or 5–10 µM 15d-PGJ_2_ were added where specified. Primary AML mononuclear cell preparations were prepared using Ficoll Paque-Plus (Pharmacia Biotech, UK) from presentation aspirates and peripheral blood samples provided after informed consent and ethical committee approval from ongoing phase I/II trials currently undertaken within the University of Birmingham Hospitals NHS Trust and from the MRC AML 15 phase III trial. The resultant AML blast preparations were cultured at 1×10^6^ cells/ml in RPMI 1640 supplemented with 1% (v/v) ITS^+^ (VWR, UK), IL3 (1 ng/ml) and SCF (10 ng/ml) (both R&D Systems, UK). For treatments, primary AML cells were set at 1×10^6^ cells/ml with either solvent control, 5 µM MPA, 0.5 mM (500 µM) BEZ or B/M, ±1 nM ATRA+D3. Viability was determined by manual cell counts and cytospin preparations made for analysis of morphology. Normal donor mobilised peripheral blood samples were provided under ethical approval and informed consent by the National Blood Service, Stem Cell Laboratories, Birmingham, UK, and treated as for primary AML cells.

### ^3^H-PGD_2_ Turnover Analysis by Thin Layer Chromatography

PGD_2_ turnover in intact cells was determined similarly to that described previously [28]. Briefly, 2×10^6^ KG1a cells were incubated with 0.2 µCi/1.3pmoles ^3^H-PGD_2_ (Amersham Biosciences) in warm PBS for 9 hours. Prostaglandin extracts were prepared from supernatants and separated on silica gel/TLC plates before reading on a Bioscan plate reader. Prostanoids were identified by their co-migration with known standards (Biomol International L.P., UK) visualized by placing in a sealed tank filled with iodine vapor for approximately 5 minutes.

### Lipid Peroxidation Assay

2×10^6^ cells at 2.5×10^5^/ml were treated at 37°C for 2 hours with the addition of 10 µM *cis*-parinaric acid (a naturally fluorescent lipid) for the last 30 mins. Cells were harvested and lipids extracted in the dark for 1 hour with isopropanol + 0.05% butylated hydroxytoluene. Fluorescence readings were taken at 320 nm_excitation_/415 nm_emission_. Background fluorescence of lipids extracted from unlabelled control cells was subtracted from all values and data presented as % fluorescence units (FU) compared to solvent control cells.

### Assessment of Accumulation of ROS

Cells were treated for 14 hours at 37°C and 10 µM carboxy-H_2_DCFDA (Molecular Probes, Invitrogen, UK) added to cells for the final 45 mins. Following incubation, cells were washed with PBS and analysed by flow cytometry (Becton Dickinson FACS Calibur and Becton Dickinson Cell Quest software).

### Prostaglandin ELISAs

Prostaglandin D2-MOX EIA kit (Cayman Chemicals, USA) and 15Δ^12,14^Prostaglandin J2 Enzyme Immunoassay Kit (Assay Designs, Inc., USA) were used to determine prostaglandin levels. 5×10^6^ cells were treated for 2 hours. Following treatment, cells were harvested with 1 ml culture media and homogenised using a Precellys 24 ceramic bead based homogenisation system. Prostaglandins were extracted using C18 reverse phase extraction columns (Chromabond, Fisher, UK) and levels determined by ELISA.

### Measurement of GSH Levels

3×10^6^ cells were treated at 2.5×10^5^/ml, cell pellets prepared and GSH levels determined as described previously [69].

### PPRE Reporter Assays

Briefly, 2×10^6^ HL-60 cells were transfected with 2.5 µg p4xACO-Luciferase (kind gift from Prof Bert Vogelstein, John Hopkins, USA) and 0.5 µg pRenilla-luciferase (Promega, UK) using Solution V and file T19 on the AMAXA Nucleofector I system. Transfected cells were allowed to recover for 14 hrs and then treated for 24 hrs. Cells were harvested and luciferase measured using the Dual-Glo® Luciferase Assay Kit (Promega, UK) according to manufacturer's instructions.

### Western Blot Analyses

Cells treated with drugs for 14 hrs were lysed in RIPA buffer and 30 µg proteins separated by SDS-PAGE. Proteins were transferred to Immobilon-P membrane (Millipore Corp, Bedford, MA, USA) and probed with 1/1000 dilution of anti-IκB (Santa Cruz, USA). Detection was by anti-rabbit-horse radish peroxidise (HRP) diluted 1/1000 and ECL using Supersignal West Pico Chemiluminescent substrate (Pierce, USA). Loading controls used anti β-actin antibody (Sigma UK) diluted 1/25000 and anti-mouse-HRP secondary at 1/25000. Densitometry was performed using ImageJ software (http://rsb.info.nih.gov/ij/) and IκB protein expression normalised to β-actin.

### Measurement of Relative Number of Viable Cells

Myeloid cell lines were treated for 7 days with refeeding every 2 days. Numbers of viable cells in triplicate 100 µl aliquots of cultures were determined using CellTiter-Blue® reagent (CellTiter-Blue® Cell Viability Assay, Promega, UK). Readings were adjusted for feeding schedules over the course of treatment.

### Assessment of Cell Differentiation

Myeloid cell lines were treated for 7 days with refeeding and retreating every 2 days. Analysis of differentiation antigen expression was by flow cytometry (Becton Dickinson FACS Calibur and Becton Dickinson Cell Quest software) using FITC CD71 and PE-CD11b (Becton Dickinson) conjugated antibodies for HL60, U937, NB4 and KG1a cells. FITC-Glycophorin-A was used to determine erythroid differentiation of K562 (Serotec, UK).

### Assessment of Apoptosis by Annexin-V

Phosphotidylserine cell surface expression was assessed using Annexin-V FITC kit (Becton Dickinson, UK). Analyses were carried out by Flow cytometry on a Becton Dickinson FACS Calibur utilising Cell Quest Pro software (Becton Dickinson, UK).

### Jenner Giemsa Staining of Slides

Cytospins were prepared from 75–100 µl of culture. Slides were air-dried, methanol fixed and stained; first with Jenner staining solution (VWR, UK) diluted 1/3 in 1 mM sodium phosphate buffer pH 5.6 (5 mins) and second with Giemsa stain (VWR, UK) diluted 1/20 in 1 mM sodium phosphate buffer pH 5.6 (10 mins). Slides were dried and then mounted onto coverslips using DePex (VWR, UK).

### Statistics

Data were analysed using SPSS v15 and the non-parametric Mann-Whitney U test. Unless stated in the legend, the statistics shown in figures are all compared to control cultures.

## Supporting Information

Figure S1 Cell viability is reduced in Myeloid cell lines treated with BEZ, MPA or B/M. Cell viability as % of solvent treated controls was determined in 5 myeloid cell lines by Alamar Blue assay following treatment with either solvent control, 0.5 mM BEZ, 5 uM MPA or the combination (B/M) for 7 days. Cell viability was calculated for treatments relative to solvent treated controls after readings had been adjusted for feeding regimens over the 7 days of treatment. Mean is indicated by the black bars. Statistics * p<0.01.
(0.12 MB PPT)

Figure S2 Effect of BEZ, MPA and B/M on myeloid cell lines. (A) Differentiation was measured by flow-cytometry using the myeloid differentiation antigen CD11b for HL60, NB4, U937, KG1a cells and the erythroid antigen Glycophorin-A for differentiation of K562. Scatter plots show data from a minimum of N = 3 experiments. Mean is indicated by black bar. (B) % Sub-G1 events were measured by flow cytometry cell cycle analysis of propidium iodide stained cells following 7 days treatment. Scatter plot shows data from a minimum of N = 3 experiments. Mean is indicated by black bar.
(0.21 MB PPT)

Figure S3 PGD_2_ synthesis, metabolism and non-enzymatic conversions towards 15dΔ^12,14^PGJ_2_. Adapted from Gao et al, 2003 (JBC 278: 28479–89). PGD_2_ is highly unstable and rapidly undergoes non-enzymatic conversions to form 15dΔ^12,14^PGJ_2_ in the absence of AKR1C3. Solid arrows and dotted arrows indicate enzyme mediated and non-enzymatic conversions respectively.
(0.09 MB PPT)

Figure S4 ROS induction in myeloid cell lines and primary AMLs. Reactive oxygen species (ROS) induction was determined by staining with carboxy-H2 DCFDA and flow cytometry in HL60, KG1a and primary AML samples after 48 hours treatment with CONT, B/M or 10 mM 15d-PGJ2. Data shown is N = 4 for HL60 and KG1a and N = 3 primary AMLs. Mean is indicated by black bar. Statistics * p<0.01
(0.06 MB PPT)

Figure S5 1H-1H 2D correlation spectroscopy (COSY) NMR spectrum of KG1a cells extracts. Expanded region (2.6–4.8 ppm) of 1H-1H 2D COSY 45 (COrrelation SpectroscopY) NMR spectrum acquired on dried polar extracts of KG1a cells (solvent control treatment) redissolved in 99.9% D2O (GOSS Scientific Instruments Ltd, Essex UK). 2D COSY experiments were carried out using 800 MHz Varian spectrometer equipped with a cryogenically cooled probe using a gradient-selected coherence transfer pathway (gCOSY45) (Hurd, John & Plant, 1991, J Mag Reson, 93: 666) with 16 transients of 8192 complex data points, 256 increments, and a spectral width of 8 kHz in both dimensions. The highlighted peaks (red lines) are due to oxidized (GSSG) and reduced (GSH) glutathione.
(0.42 MB PPT)

Figure S6 1H 1D NMR spectrum of KG1a cells extracts. Cells were treated for 24 hours and polar extracts analysed by NMR. Extraction of metabolites from cells pellets was performed using a modified Bligh-Dyer procedure. Dried polar extracts were redissolved in 90% H_2_O/10% D_2_O (GOSS Scientific Instruments Ltd, Essex UK) with phosphate buffer (100 mM, pH 7), containing 0.5 mM TMSP. A 500 MHz Bruker spectrometer equipped with a cryogenically cooled probe was used for 1D 1H data acquisition. The water resonance was suppressed using excitation sculpting (Hwang & Shaka, 1998, J Magn Reson, 135: 280). 1D spectra were acquired using a 60° pulse, a 5 kHz spectral width, a relaxation delay of 3 s with 128 transients. 3 different sections (2.15–2.22, 2.50–2.62, and 2.95–3.02 ppm) of the 1H 1D NMR spectrum of KG1a cell extracts containing glutathione peaks. A minimum of 12 replicates for each treatment (black, solvent control; red, MPA, green BEZ, blue B/M) are shown. The insert depicts the average spectrum of 12 replicates expanded between 2.15–2.22 ppm.
(0.22 MB PPT)

Figure S7 I-kappa B levels are reduced in some myeloid cell lines following treatment with B/M. IkB levels were determined by western blotting of cells treated with either solvent control, 0.5 mM BEZ, 5 µM MPA or the combination for 14 hrs. Levels were normalised for loading by β-actin westerns and densitometry. Scatter plots show all datapoints for a minimum of N = 3 experiments for each cell lines. Means are indicated by black bars. Statistics *p<0.05
(0.11 MB PPT)

Figure S8 Model of B/M action against AML cells. ROS directly generated by BEZ and indirectly by subsequently generated 15-deoxy-Δ^12,14^PGJ_2_, enhances PGD_2_ production via the lipid peroxidation isoprostane pathway. Inhibition of AKR1C3 by MPA results in diversion of PGD_2_ towards the J series prostaglandins culminating in the pleiotropic anti neoplastic actions of 15-deoxy-Δ^12,14^PGJ_2_ including further generation of ROS and activation of lipid peroxidation.
(0.10 MB PPT)
