# Supplementary figures and images for: Combined Bezafibrate and Medroxyprogesterone Acetate: Potential Novel Therapy for Acute Myeloid Leukaemia
Source: PLoS One. 2009 Dec 7;4(12):e8147. doi: 10.1371/journal.pone.0008147 (PMC2785482; doi:10.1371/journal.pone.0008147)

## Slide 1
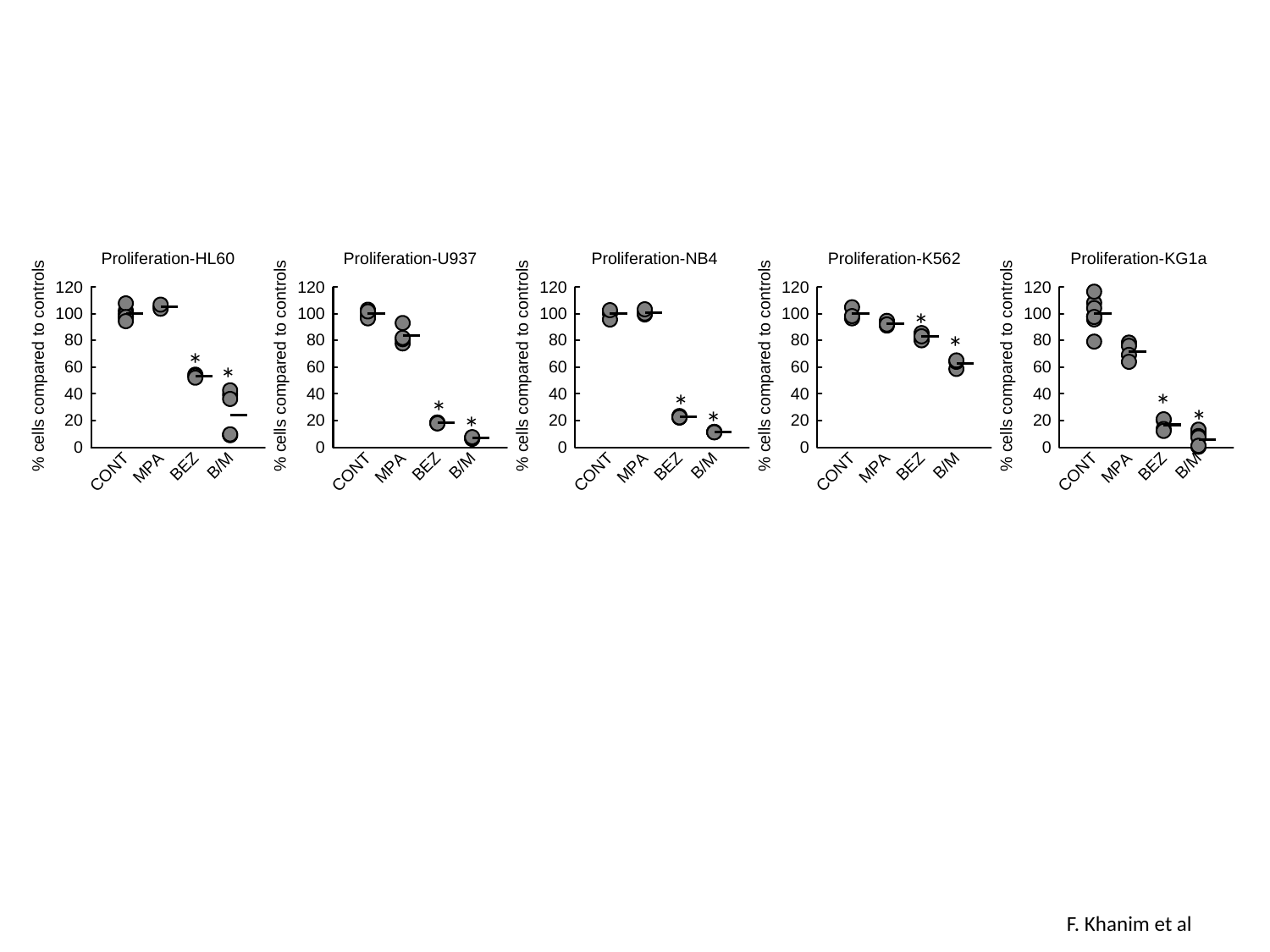

*
*
*
*
*
*
*
*
*
*
F. Khanim et al

Supplement: Figure S1 — Cell viability is reduced in Myeloid cell lines treated with BEZ, MPA or B/M. Cell viability as % of solvent treated controls was determined in 5 myeloid cell lines by Alamar Blue assay following treatment with either solvent control, 0.5 mM BEZ, 5 uM MPA or the combination (B/M) for 7 days. Cell viability was calculated for treatments relative to solvent treated controls after readings had been adjusted for feeding regimens over the 7 days of treatment. Mean is indicated by the black bars. Statistics * p<0.01. (0.12 MB PPT) [file pone.0008147.s001.ppt]

## Slide 1
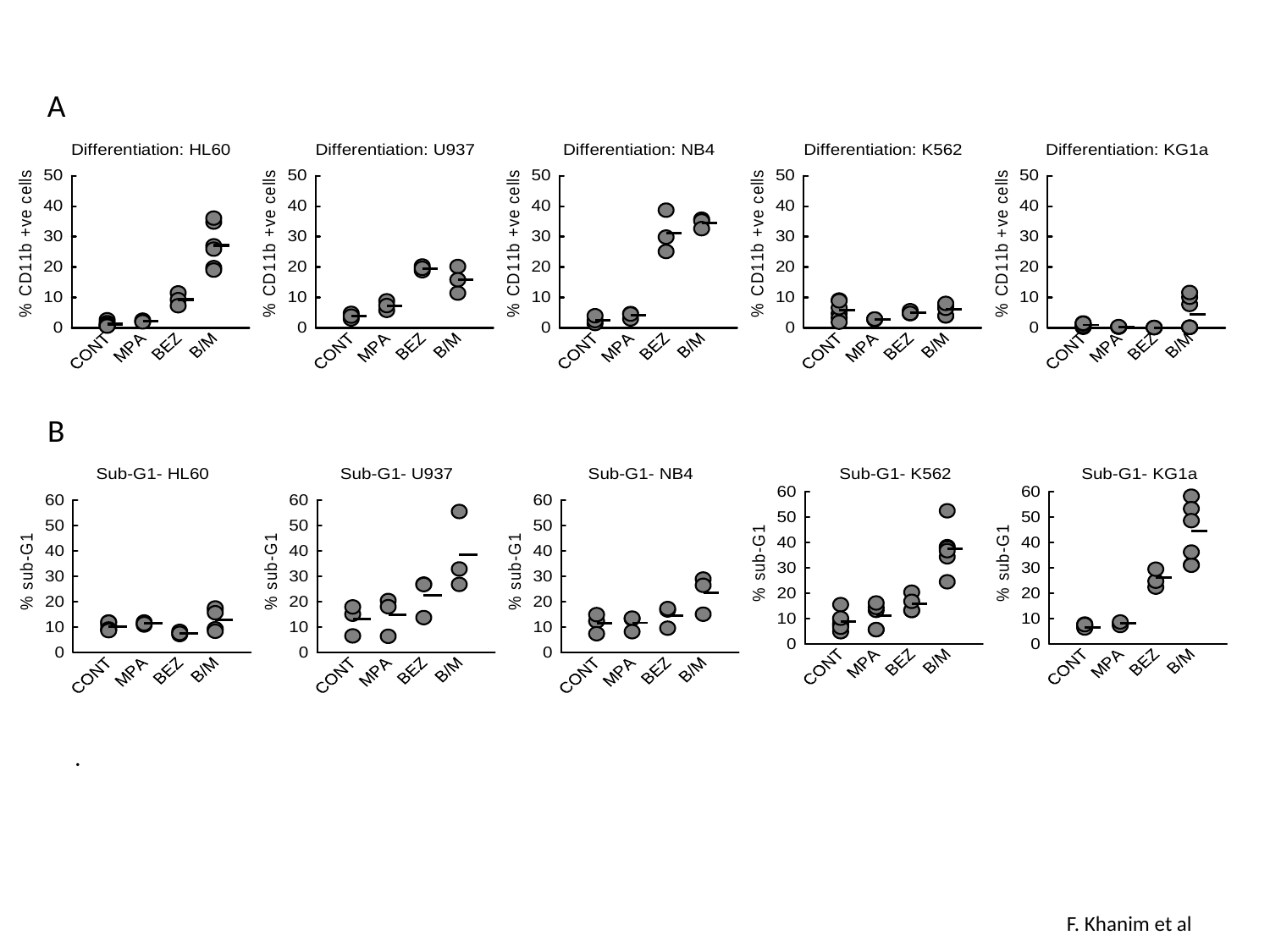

A
B
.
F. Khanim et al

Supplement: Figure S2 — Effect of BEZ, MPA and B/M on myeloid cell lines. (A) Differentiation was measured by flow-cytometry using the myeloid differentiation antigen CD11b for HL60, NB4, U937, KG1a cells and the erythroid antigen Glycophorin-A for differentiation of K562. Scatter plots show data from a minimum of N = 3 experiments. Mean is indicated by black bar. (B) % Sub-G1 events were measured by flow cytometry cell cycle analysis of propidium iodide stained cells following 7 days treatment. Scatter plot shows data from a minimum of N = 3 experiments. Mean is indicated by black bar. (0.21 MB PPT) [file pone.0008147.s002.ppt]

## Slide 1
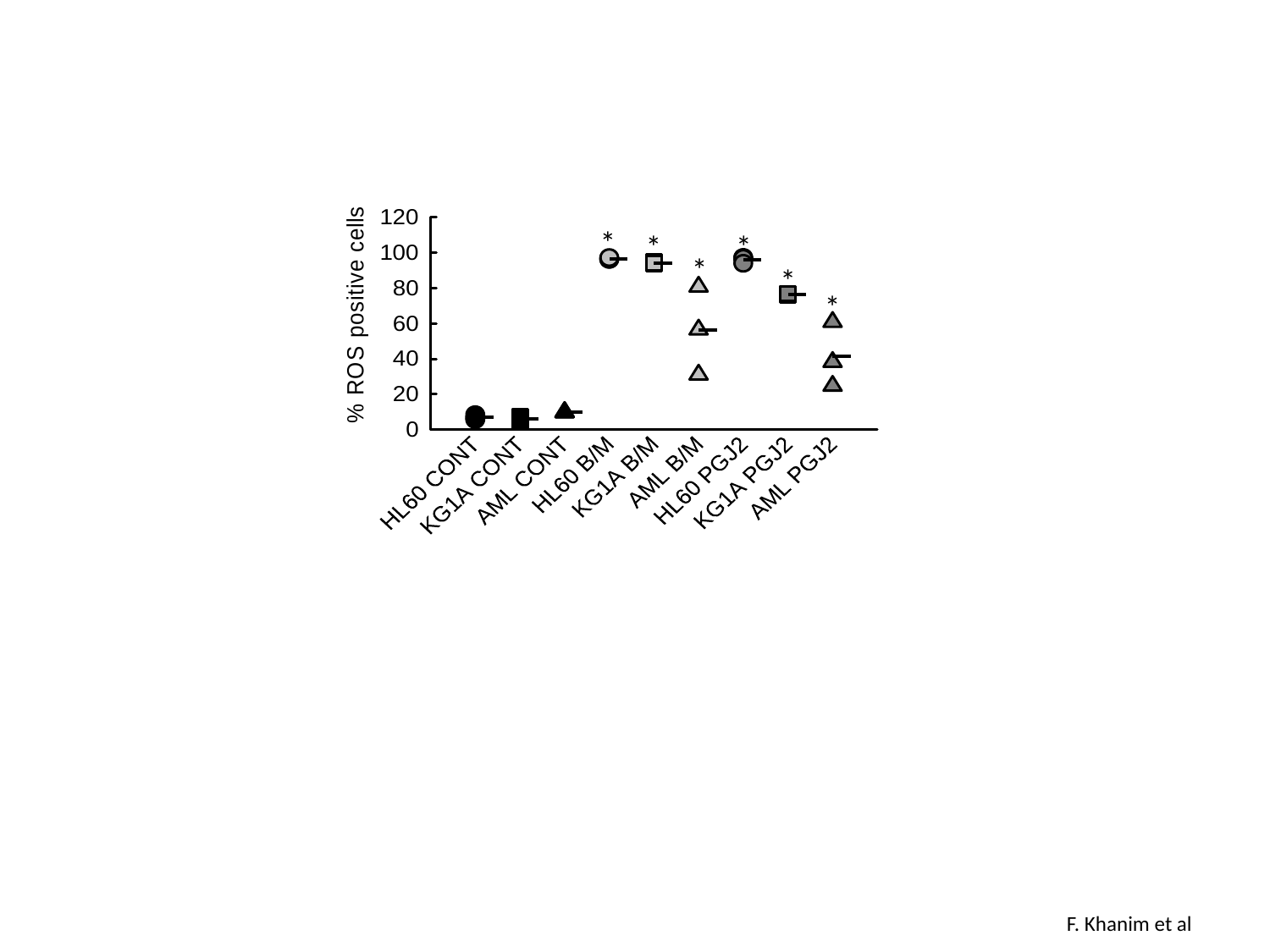

*
*
*
*
*
*
F. Khanim et al

Supplement: Figure S4 — ROS induction in myeloid cell lines and primary AMLs. Reactive oxygen species (ROS) induction was determined by staining with carboxy-H2 DCFDA and flow cytometry in HL60, KG1a and primary AML samples after 48 hours treatment with CONT, B/M or 10 mM 15d-PGJ2. Data shown is N = 4 for HL60 and KG1a and N = 3 primary AMLs. Mean is indicated by black bar. Statistics * p<0.01 (0.06 MB PPT) [file pone.0008147.s004.ppt]

## Slide 1
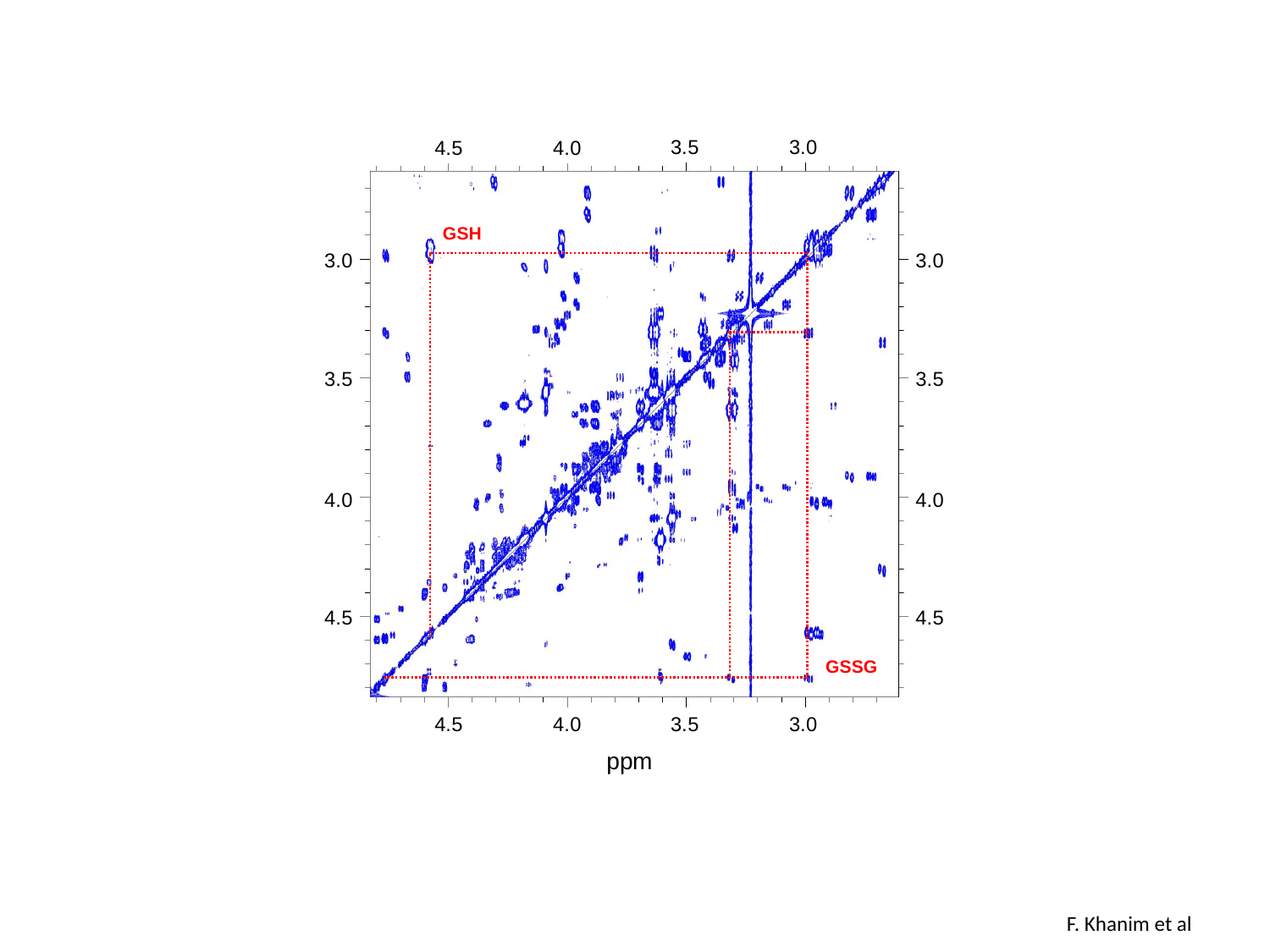

F. Khanim et al

Supplement: Figure S5 — 1H-1H 2D correlation spectroscopy (COSY) NMR spectrum of KG1a cells extracts. Expanded region (2.6–4.8 ppm) of 1H-1H 2D COSY 45 (COrrelation SpectroscopY) NMR spectrum acquired on dried polar extracts of KG1a cells (solvent control treatment) redissolved in 99.9% D2O (GOSS Scientific Instruments Ltd, Essex UK). 2D COSY experiments were carried out using 800 MHz Varian spectrometer equipped with a cryogenically cooled probe using a gradient-selected coherence transfer pathway (gCOSY45) (Hurd, John & Plant, 1991, J Mag Reson, 93: 666) with 16 transients of 8192 complex data points, 256 increments, and a spectral width of 8 kHz in both dimensions. The highlighted peaks (red lines) are due to oxidized (GSSG) and reduced (GSH) glutathione. (0.42 MB PPT) [file pone.0008147.s005.ppt]

## Slide 1
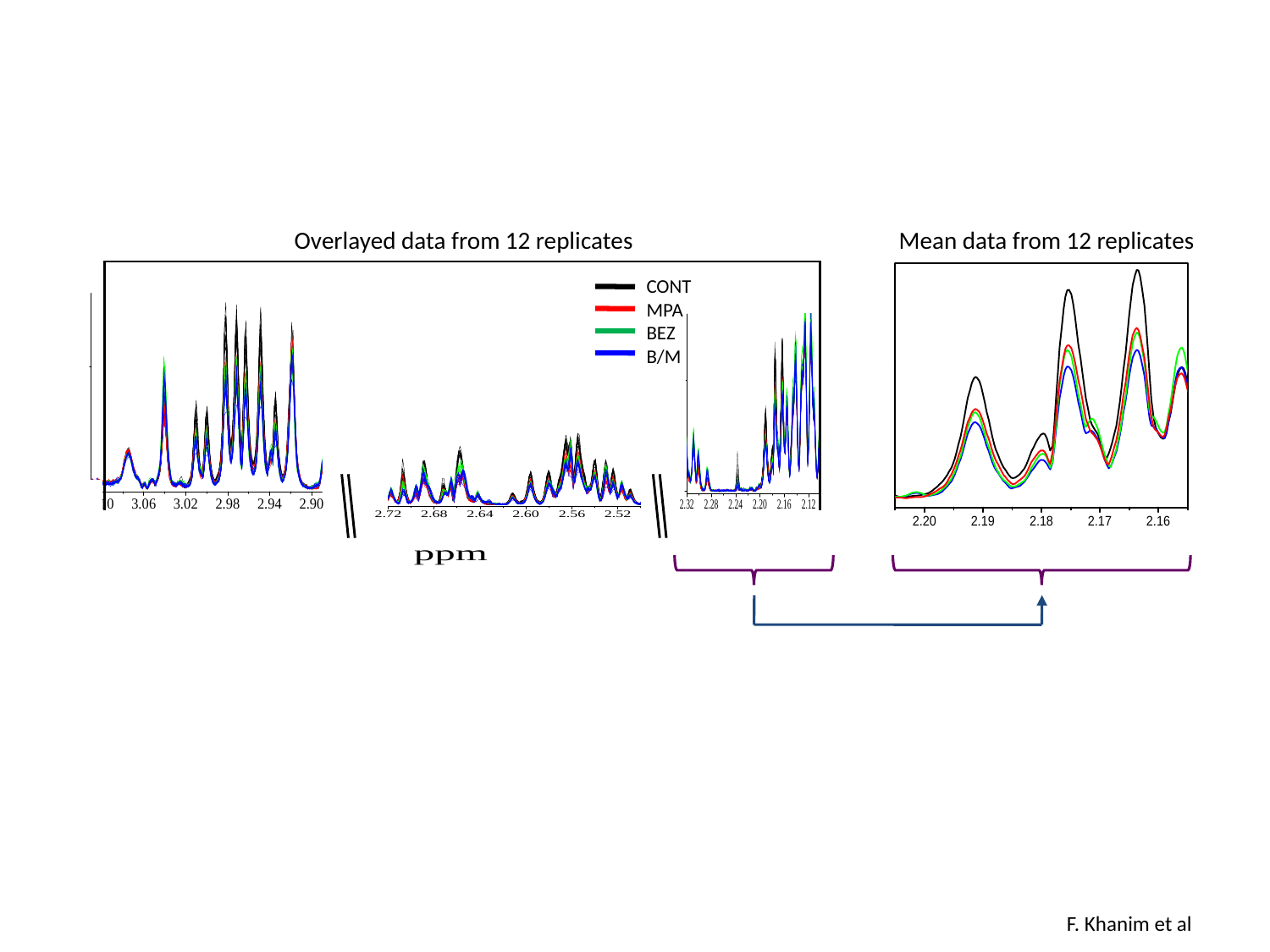

Overlayed data from 12 replicates
Mean data from 12 replicates
CONT
MPA
BEZ
B/M
F. Khanim et al

Supplement: Figure S6 — 1H 1D NMR spectrum of KG1a cells extracts. Cells were treated for 24 hours and polar extracts analysed by NMR. Extraction of metabolites from cells pellets was performed using a modified Bligh-Dyer procedure. Dried polar extracts were redissolved in 90% H2O/10% D2O (GOSS Scientific Instruments Ltd, Essex UK) with phosphate buffer (100 mM, pH 7), containing 0.5 mM TMSP. A 500 MHz Bruker spectrometer equipped with a cryogenically cooled probe was used for 1D 1H data acquisition. The water resonance was suppressed using excitation sculpting (Hwang & Shaka, 1998, J Magn Reson, 135: 280). 1D spectra were acquired using a 60° pulse, a 5 kHz spectral width, a relaxation delay of 3 s with 128 transients. 3 different sections (2.15–2.22, 2.50–2.62, and 2.95–3.02 ppm) of the 1H 1D NMR spectrum of KG1a cell extracts containing glutathione peaks. A minimum of 12 replicates for each treatment (black, solvent control; red, MPA, green BEZ, blue B/M) are shown. The insert depicts the average spectrum of 12 replicates expanded between 2.15–2.22 ppm. (0.22 MB PPT) [file pone.0008147.s006.ppt]

## Slide 1
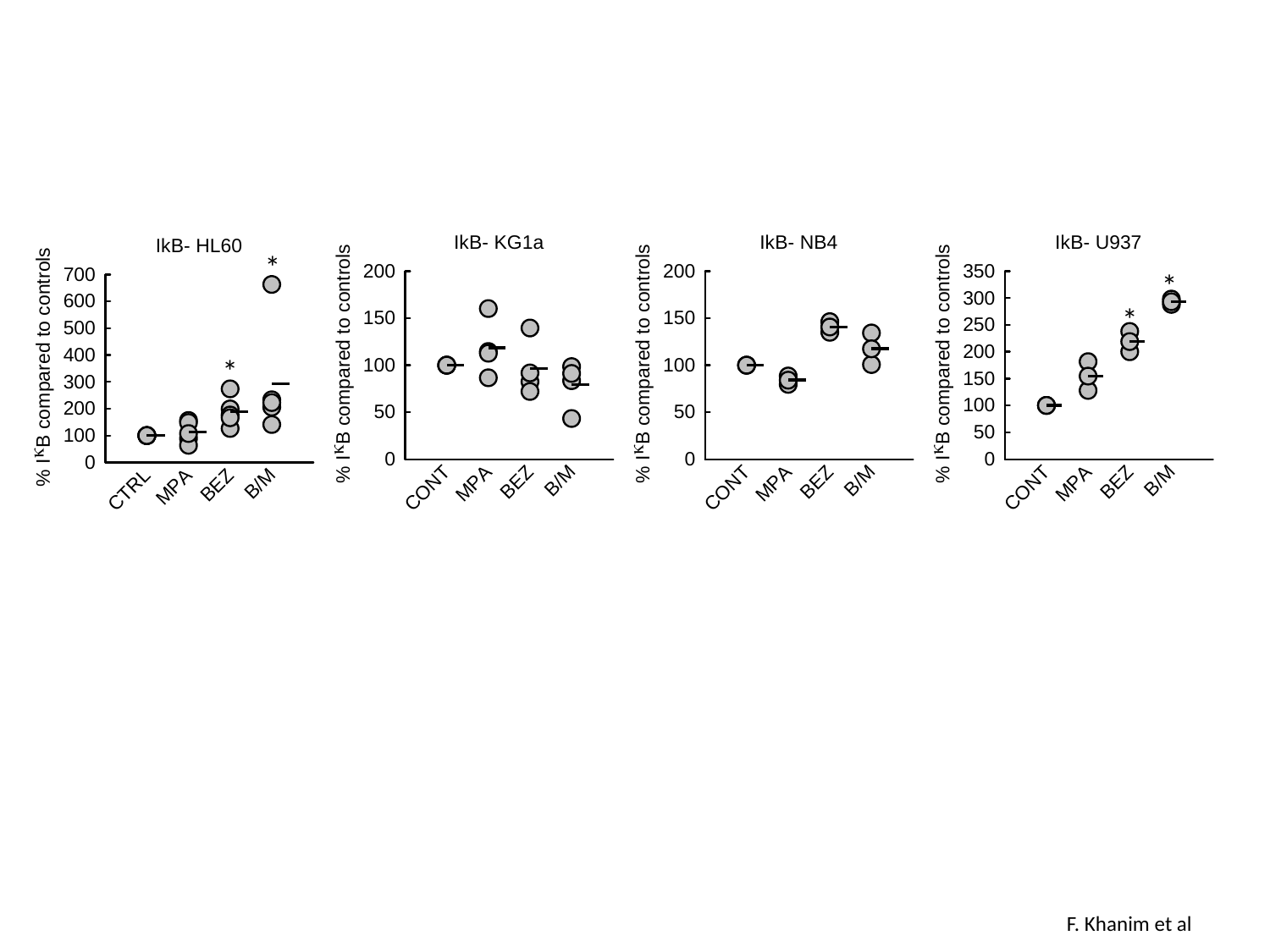

*
*
*
*
F. Khanim et al

Supplement: Figure S7 — I-kappa B levels are reduced in some myeloid cell lines following treatment with B/M. IkB levels were determined by western blotting of cells treated with either solvent control, 0.5 mM BEZ, 5 µM MPA or the combination for 14 hrs. Levels were normalised for loading by β-actin westerns and densitometry. Scatter plots show all datapoints for a minimum of N = 3 experiments for each cell lines. Means are indicated by black bars. Statistics *p<0.05 (0.11 MB PPT) [file pone.0008147.s007.ppt]
