# Supplementary material for: Combined Bezafibrate and Medroxyprogesterone Acetate: Potential Novel Therapy for Acute Myeloid Leukaemia
Source: PLoS One. 2009 Dec 7;4(12):e8147. doi: 10.1371/journal.pone.0008147 (PMC2785482; doi:10.1371/journal.pone.0008147)
Supplement: Figure S8 — Model of B/M action against AML cells. ROS directly generated by BEZ and indirectly by subsequently generated 15-deoxy-Δ12,14PGJ2, enhances PGD2 production via the lipid peroxidation isoprostane pathway. Inhibition of AKR1C3 by MPA results in diversion of PGD2 towards the J series prostaglandins culminating in the pleiotropic anti neoplastic actions of 15-deoxy-Δ12,14PGJ2 including further generation of ROS and activation of lipid peroxidation. (0.10 MB PPT) [file pone.0008147.s008.ppt]

## Slide 1
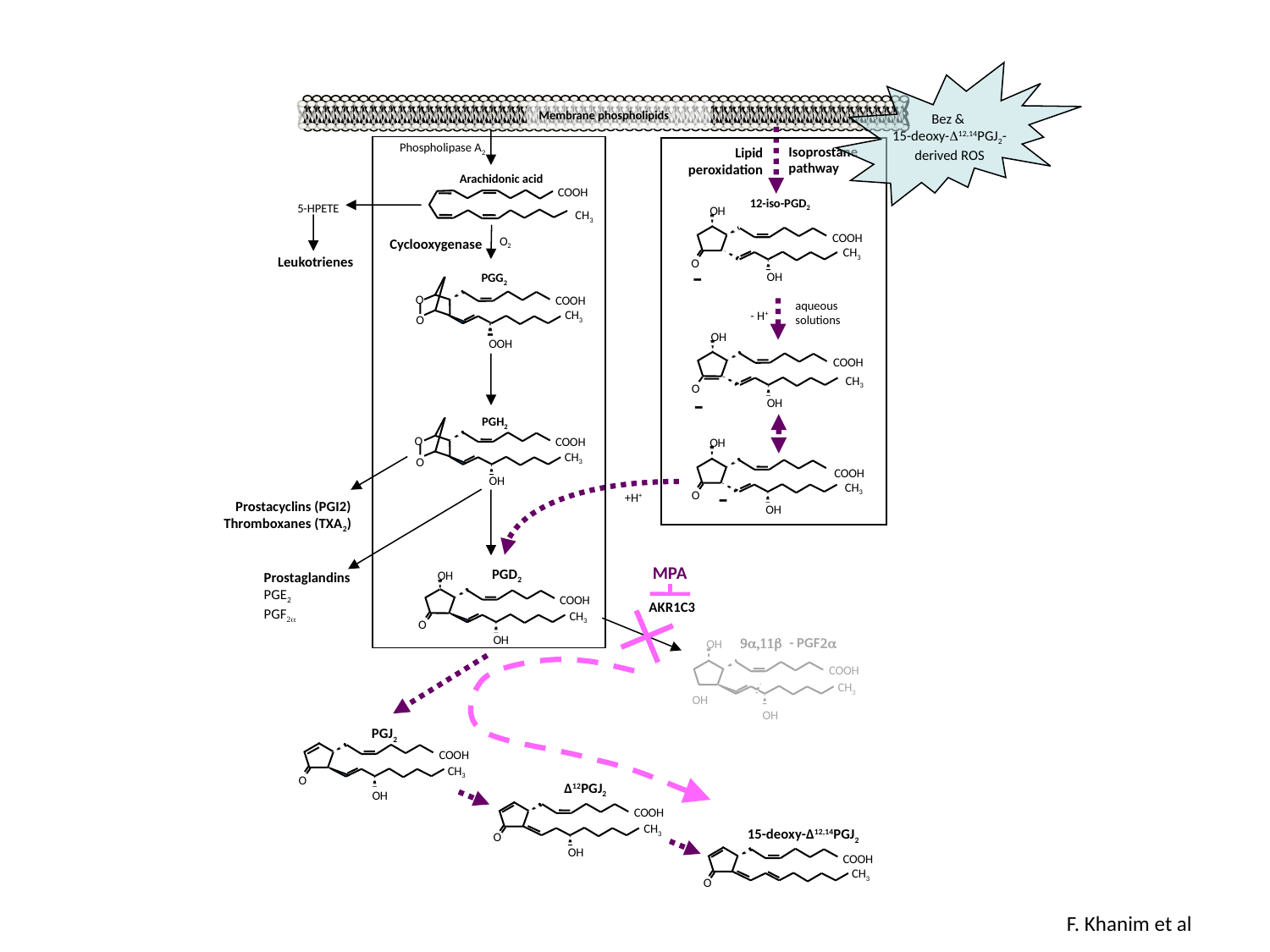

Bez &
15-deoxy-Δ12,14PGJ2-
derived ROS
Membrane phospholipids
Phospholipase A2
Isoprostane
pathway
Lipid
peroxidation
Arachidonic acid
COOH
12-iso-PGD2
5-HPETE
OH
COOH
CH3
O
-
OH
CH3
O2
Cyclooxygenase
Leukotrienes
PGG2
O
COOH
aqueous
solutions
CH3
- H+
O
OH
COOH
CH3
O
-
OH
OOH
PGH2
O
COOH
OH
COOH
-
CH3
O
OH
CH3
O
OH
+H+
Prostacyclins (PGI2)
Thromboxanes (TXA2)
MPA
PGD2
COOH
CH3
O
OH
OH
Prostaglandins
PGE2
PGF
AKR1C3
 - PGF
COOH
CH3
OH
OH
OH
PGJ2
COOH
CH3
O
OH
Δ12PGJ2
COOH
CH3
O
OH
15-deoxy-Δ12,14PGJ2
COOH
CH3
O
F. Khanim et al
